# Public perceptions of government policies to COVID-19: A comparative study in six African countries

**DOI:** 10.1016/j.heliyon.2024.e24888

**Published:** 2024-01-24

**Authors:** Yi-jun WANG, Marly Loria DIABAKANGA BATATANA, Maximino Horacio BIKOUMOU GAMBAT

**Affiliations:** a School of Public Administration, Xi'an University of Architecture and Technology, Shaanxi, 710055, China; b School of Civil Engineering, Chang'an University, Shaanxi, 710064, China

**Keywords:** Public perceptions, Government policies, COVID-19, Twitter, Sentiment analysis

## Abstract

The outbreak of COVID-19 has affected countries across the world, including those in Africa. Governments in these countries have implemented various policies to curb the spread of the virus. However, the effectiveness of these policies largely depends on how the public perceives them. This study aims to investigate public perceptions of government policies regarding COVID-19 in six African countries by conducting a sentiment analysis of the public. The motivation behind this study relies in the recognition that a deeper understanding of public perceptions is essential for crafting effective strategies that resonate with the diverse needs and concerns of the population, ultimately contributing to the ongoing global efforts to navigate the complexities of the COVID-19 pandemic. We collected tweets related to COVID-19 and government policies on Twitter's API from March 07, 2020 to February 02, 2022. We performed data processing steps such as tokenization and stop-word removal to clean the data. Next, we used Natural Language Processing (NLP) techniques to classify the sentiment of each tweet as positive, negative, or neutral. The six African countries selected for this study are Nigeria, South Africa, Kenya, Ghana, Rwanda, and Uganda. We collected 134,494 tweets on Twitter accounts and we evaluated policies by countries in three categories: while some countries implemented too strict policies, others implemented strict or relaxed policies. The findings of this study will provide valuable insights into how the public perceived the policies. This is used to advice policymakers and public health officials on enhancing their messaging and policies to combat the spread of COVID-19 effectively. Data showed heterogeneous reactions with negative perceptions, for instance, earlier, different governments implemented face mask and lockdown policies and vaccination policy later. Researchers and policymakers should exercise caution and consider complementary data sources and methods to ensure a more comprehensive and accurate understanding of public perceptions in the context of government policies related to COVID-19; also, investigate how government policies during the pandemic may have affected the environment, such as changes in pollution levels, waste management, and conservation efforts.

## 1 Introduction

The world is sometimes upset by situations that can be restrictive for human beings [1]. COVID-19 is nowadays one of the cases that change the world on all sides of life. COVID-19, a new disease, appeared for the first time in Wuhan city at the end of 2019 [2]. However, the cause of its apparition is unknown [3]. The World Health Organization announced COVID-19 as a pandemic on January 30, 2020, and within three months, the virus had spread almost worldwide [4]. In Africa, most of the diagnosed imported cases arrived from Europe and the United States as an alternative to China, the origin of the virus. The signs and symptoms attack the body's immune system, thereby inflicting extreme fatalities on the physique. COVID-19 is a novel disease; it was difficult to find a drug to treat it. Moreover, the World Health Organization and clinical specialists recommended that the fundamental remedy is to stay away from physical contact. In addition, do not move from one location to another, take vaccine and wear masks. The disease put the continent in a very difficult situation. The implementation of the first lockdown was in Wuhan on January 23, 2020. In Africa, the first case found in Nigeria on February 27, 2020, was an Italian who worked in Nigeria and came from Milan on February 25, 2020. Within a few days, the virus was spread to the rest of the countries, like South Africa, on March 5, 2020 considered as the most country touched by the pandemic in Africa, Ghana on March 12, 20220, Kenya on March 13, 2020, Rwanda on March 14, 2020, and Uganda on March 29, 2020. In early April 2020, about 3.9 billion people were in some form of lockdown. African countries entered the pandemic with different degrees of confidence. The connection between COVID-19, face masks, lockdowns, vaccines, and the environment in Africa is multifaceted while efforts to control the spread of the virus have had environmental consequences; there is a growing awareness of the need for sustainable practices in healthcare and waste management. Balancing public health priorities with environmental considerations is crucial for a comprehensive and sustainable response to the ongoing challenges posed by the pandemic.

Our study aimed to take four following factors together: we analysed public perceptions of COVID-19 policies implemented by six African governments for comparison, but several existing studies investigated the public perceptions of COVID-19 policies using two or three countries. Furthermore, we explain the reasons for numerous negative perceptions. Additionally, compared to the previous study, our period of the analysis is longer. This period helped us to collect many tweets and to better understand these countries. Finally, we also analysed three policies, in contrary to previous research, which highlighted one or two of the policies we chose [5]. The remainder of this research is structure as follows: in the next section, we explain our methodology and data collection in section 2, the result and discussion in section 3, and the conclusion in section 4.

## 2 Data and methodology

### 2.1 Methodology

In this section, we explained how we collected data for our experiment. We use Twitter as a social media platform. Considering the importance of social media for influencing public trust in government. Governments of countries in Africa have initiated several programs on how to use social media to communicate with their citizens [6]. We selected Twitter as the primary social media platform for our data collection due to its widespread usage and influence on public discourse, especially regarding government-related topics. This choice aligns with the growing recognition of social media's impact on public perception, as highlighted [7]. As a response to this, several African governments have initiated programs to leverage social media platforms like Twitter as a means of communication with their citizens. To understand people's opinions regarding their trust in government, we employed sentiment analysis techniques [8].

### 2.2 Data sources

The data comes from citizens’ comments on Twitter accounts and coded in the Python programming language to conduct the sentiment analysis. The data was annual. We collected 134,494 tweets from Twitter accounts. The collection period was from March 7, 2020, to February 28; 2022. The choice of this period is justified by the fact that the first case found in Africa was in February 2020. Additionally, this period was the most difficult for African countries. We retrieved data with #lockdown# face mask#, and #vaccine as keywords. We used three main steps to proceed with the data by country. In the first step, we scraped data using Python libraries on Google Collab, we restricted our filter to the English language, and limited tweets to 100.000 tweets maximum. We also mention the name, region, category of policy, and the start date and the end date. The second step was the data-cleaning process. We began by inspecting the raw data to understand its structure, formats, and potential issues; examine the first few rows, data types, and summary statistics to get a sense of the dataset. Identify and handle missing values. This involved removing rows and columns with a high percentage of missing values; the raw data generated tweets that help us to find clarity in the data processing. The score of our confidence was 0.5. We used tokenization, which is a crucial step in natural language processing (NLP) and machine-learning tasks involving conversion of tweets into numerical features and the stop words removal that are words that no longer alternate the meaning of the information in the corpus. In essence, by eliminating such phrases from the text, the subject extraction algorithm ought to take as input, the most vital words producing more results. We focus on the most important words: “face mask”, “lockdown”, “and vaccine”. Once we tokenized the data, each token assigned a unique index or identifier. In the third step, we made a sentiment analysis with the lexicon ratings defined with values of negative 1, positive 2, and neutral 0. The result saved in a comma-separated value (CSV) file. This helped us to create visualizations with charts and pie to represent sentiment distributions using Origin pro software by selecting relevant information such as: the name of the country, the date of data collection, and the confidence of tweets. To make a better comparison of policies in six African countries, we analysed each policy according to its respective date of implementation.

Collecting data for a comparative study of netizen perceptions requires a methodical approach. Researchers need to identify the social media platforms most popular in each of the six African countries under investigation. Using Application Programming Interface (APIs) provided by these platforms, researchers could collect a vast sample of tweets, posts, and comments that mention keywords relevant to COVID-19 policies. These keywords should encompass terms related to lockdowns, mask mandates, and vaccination campaigns. Collecting data for a comparative study of netizen perceptions requires a well-structured and systematic approach. Researchers must identify the most popular social media platforms in each of the six African countries under investigation, taking into account regional preferences.

**Fig. 1:**
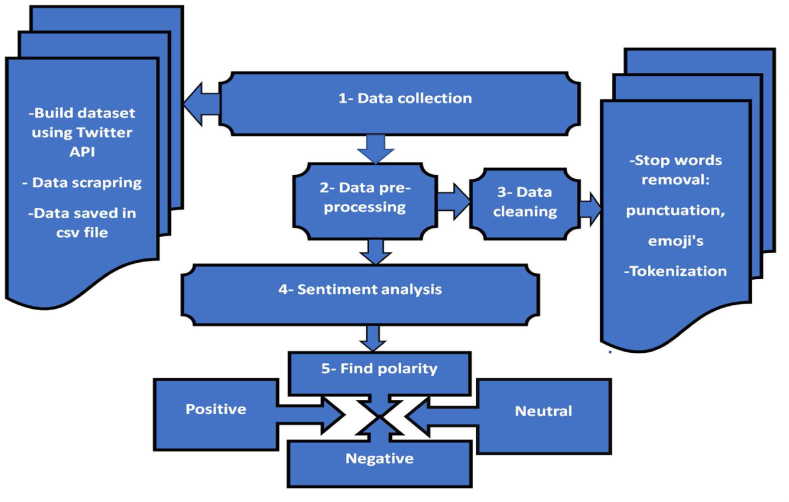
Research design. Source: Authors personal data

Fig. 1 summarizes the data collection and processing. This diagram illustrates a clear visual representation of the research design for data collection in sentiment detection. It outlines the key steps involved in gathering, preparing, and analysing the data, and the logical progression from data sources to the final sentiment detection model, emphasizing the importance of data pre-processing, labelling, and model evaluation. This visual guide serves as a helpful reference for anyone interested in understanding the methodology behind the sentiment detection study.

## 3 Results and discussion

### 3.1 Results

This study helped us to identify how social media plays an important role in detecting citizen opinion according to the measures implemented by their government to combat the COVID-19 pandemic. We analysed the government response to COVID-19 to know the perceptions of the citizens about the following measures such as face mask, lockdown, and vaccination. We showed the distribution of three levels of perceptions: positive, negative, and neutral in different countries. To show the difference between countries about all policies in Ghana, Kenya, Nigeria, Rwanda, South Africa, and Uganda during the Covid-19 pandemic, we conducted different analyses based on the number of negative tweets then we separated them into three categories; countries with too strict measures together, those with strict measures also in one group, and with relaxed measures in another. We are going to focus on the negative result to make the differences for a better explanation. We presented the results of the research in two phases: in the first phase we analysed the figures for each policy showing year-by-year results for 2020, 2021, and 2022 in 6 countries; in the second phase of our result, we are going to show different categories of measures grouped in two countries.

Table 1 presents a complete view of Twitter exercise in a variety of countries, allowing for comparisons and insights into regional trends. The variety of tweets offers a sense of the number of online conversations, whilst the common tweets per person can point out personal engagement. It confirmed that certain nations are extra active on Twitter. Overall, this table offers valuable insights into the dynamics of Twitter utilization on a world scale. It contains data for six African countries (Nigeria, South Africa, Kenya, Uganda, Ghana, and Rwanda) for the years 2020, 2021, and 2022. These columns represent the number of tweets related to lockdowns, vaccines, and face masks, respectively。 The numbers vary across countries and years, indicating the volume of discussion on these topics in each country during each year. Columns Tweet lockdown, Tweet face mask, and Tweet vaccine represent the number of tweets collected in different countries, Population 15–64: This represents the population aged 15–64 in each country. This is an important demographic group, often considered as the working-age population.

**Table 1:** Statistics of tweets generated in different countries.

Countries name	Years	Tweets lockdown	Tweets vaccine	Tweets face mask	Population 15–64	Percentage of population tweets *lockdown*	Percentage of population tweets vaccine	Percentage of population tweets face mask
Nigeria	2020	36690	10727	4143	111445958	0.032922	0.009625	0.003717
South Africa	2020	32715	6951	3195	38374016	0.085253	0.018114	0.008326
Kenya	2020	1698	5835	5667	30268153	0.00561	0.019278	0.018723
Uganda	2020	8223	1627	1939	23385723	0.035162	0.006957	0.008291
Ghana	2020	6097	9553	1422	18990581	0.032105	0.050304	0.007488
Rwanda	2020	1903	287	431	7568496	0.025144	0.003792	0.005695
Nigeria	2021	3893	19833	733	114665538	0.003395	0.017296	0.000639
South Africa	2021	7441	50459	2095	38815753	0.01917	0.129996	0.005397
Kenya	2021	1827	12661	1199	31146668	0.005866	0.04065	0.00385
Uganda	2021	4727	6411	681	24362660	0.019403	0.026315	0.002795
Ghana	2021	723	8137	444	19459598	0.003715	0.041815	0.002282
Rwanda	2021	473	1799	95	7803465	0.006061	0.023054	0.001217
Nigeria	2022	169	1037	39	117970520	0.000143	0.000879	0.000033
South Africa	2022	467	3591	417	39264163	0.001189	0.009146	0.001062
Kenya	2022	83	495	101	32046684	0.000259	0.001545	0.000315
Uganda	2022	407	381	51	25290128	0.001609	0.001507	0.000202
Ghana	2022	37	95	40	19920727	0.000186	0.000477	0.000201
Rwanda	2022	25	269	23	8036935	0.000311	0.003347	0.000286

To assess how the number of tweets relates to the population in each of the six countries, we obtain the total population of each country, and we collect data on the number of tweets related to COVID-19 policies in each country. After, we calculated the percentage of the population aged 15–64 represented by the number of citizens who tweeted about the COVID-19 policies. This gives us an idea of whether the tweets are a significant or a relatively small proportion of the overall population. For each country, we calculate the percentage of the population represented by the number of citizens who tweeted. Here is the formula:

$$\text{Percentage}\;\text{of}\;\text{Population}=\frac{Number\;of\;tweets}{Total\;population}\;x100$$

Interpretation and observations show vaccine: tweets South Africa consistently has the highest per capita tweets about vaccines across all years. Ghana also shows relatively high levels of discussion on vaccines. Nigeria has lower per capita vaccine tweets compared to South Africa and Ghana. Lockdown and Face Mask Tweets: Generally, the numbers for lockdown and face mask-related tweets are low compare to vaccine tweets. Kenya and Uganda have shown relatively higher per capita tweets about face masks in some years. The numbers for tweets in all categories appear to fluctuate across the years, suggesting dynamic public interest in these topics. Considerations show while the data shows the volume of tweets, it does not provide information on the sentiment or context of these tweets. Analysing sentiment could provide a more nuanced understanding. External factors such as government policies, events, or COVID-19 statistics may influence the trends observed in the data. Overall, this table provides insights into Twitter discussion patterns regarding lockdowns, vaccines, and masks in the six specified countries over the years.

#### 3.1.1 Face mask policy

Table 2 explains the trends across these African countries and suggest variations in public sentiment on Twitter over the years, with some countries experiencing declines in positive sentiment but improvements in negative sentiment. A range of factors, including political events, socio-economic conditions, and changes in online behavior, may influence these changes.

**Table 2:**
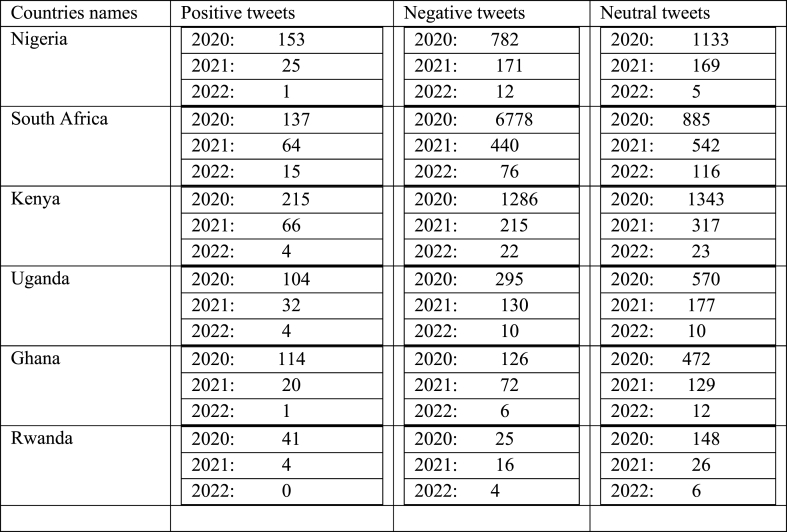
Face mask sentiment analysis of tweets by country.

Table 3 shows the regression model appears to have rational results, as indicated by the high R forecourt and the statistical significance of the F- statistic. The intercept and the measure for face mask positive tweets are statistically significant, suggesting that these variables play a part in prognosticating the dependent variable. Still, the portions for face mask negative tweets and face mask-neutral tweets are not statistically significant, indicating that these variables may not have a significant impact on the dependent variable in this model.

**Table 3:** Regression analysis: impact of face mask tweets on dependent variable.

Regression Statistics								
Multiple R	0.843136							
R Square	0.710878							
Adjusted R Square	0.648923							
Standard Error	0.497814							
Observations	18							
ANOVA								
	*Df*	*SS*	*MS*	*F*	*Significance F*			
Regression	3	8.530537	2.843512	11.47416	0.000459			
Residual	14	3.469463	0.247819					
Total	17	12						
	*Coefficients*	*Standard Error*	*t Stat*	*P-value*	*Lower 95 %*	*Upper 95 %*	*Lower 95.0 %*	*Upper 95.0 %*
Intercept	2021.615	0.158305	12770.39	7.2E-51	2021.276	2021.955	2021.276	2021.955
Face mask positive tweets	−0.02449	0.008981	−2.72623	0.016392	−0.04375	−0.00522	−0.04375	−0.00522
Face mask negative tweets	−3.5E-05	8.93E-05	−0.39157	0.70127	−0.00023	0.000157	−0.00023	0.000157
Face mask-neutral tweets	0.002285	0.001428	1.600069	0.1319	−0.00078	0.005348	−0.00078	0.005348

**Fig. 2:**
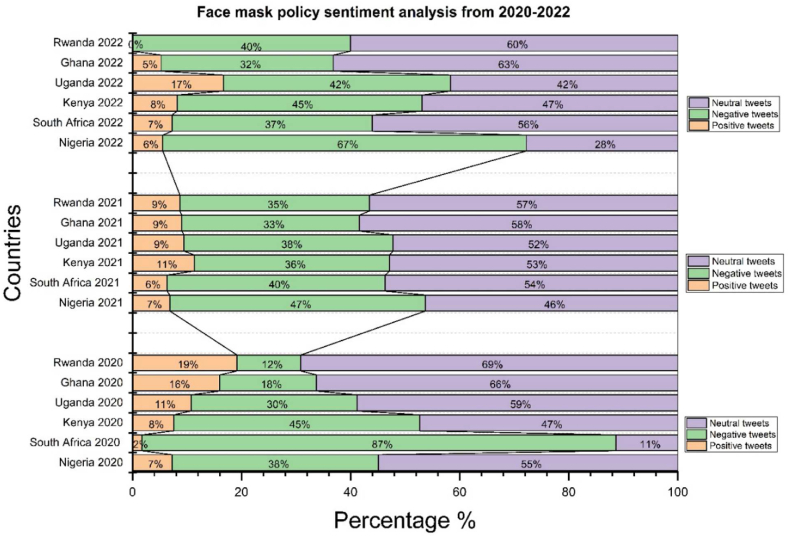
Face mask policy from 2020 to 2022 in all six countries. Source: Authors personal data

Fig. 2 explained how the face mask policy was spread in all six African countries. It shows how the tweets with positive, negative, and neutral perceptions vary over the two years. For example, the blue line represents the number of tweets with negative perceptions, the orange line represents the number with neutral perceptions and the grey line represents the number with positive perceptions. As seen in the clustered column, Kenya and Nigeria's results from 2020 to 2022 did not remain the same; as shown by the result per year, we got more reactions in 2020 than in 2021 and 2022. We tend to see a high number of tweets in 2020. This high threshold is because the C0vid-19 disease was recently and the government implemented the measure. Our results also show that from 2020 to 2022, most of the opinions were negative or neutral, but the pick of neutral perceptions every year is higher than the negative perception and few tweets are positive. On the contrary, people expressed themselves positively in 2020, but in 2021 and 2022, the trend regressed. In South Africa and Uganda, we found fewer tweets compared to Nigeria and Kenya. Our analysis also shows how many people expressed negative opinions about wearing masks in March 2020, which gives the impression that the vast majority of people in these countries are against wearing masks. The collection of most comments were in 2020, as; the information was new to them. Rwanda and Ghana: people did not react as the government expected the percentage to be very low because of a lack of information and a lack of mass in the country. In Ghana, there are more neutral and negative reactions than positive ones. There is a high pick in 2020.

**Fig. 3:**
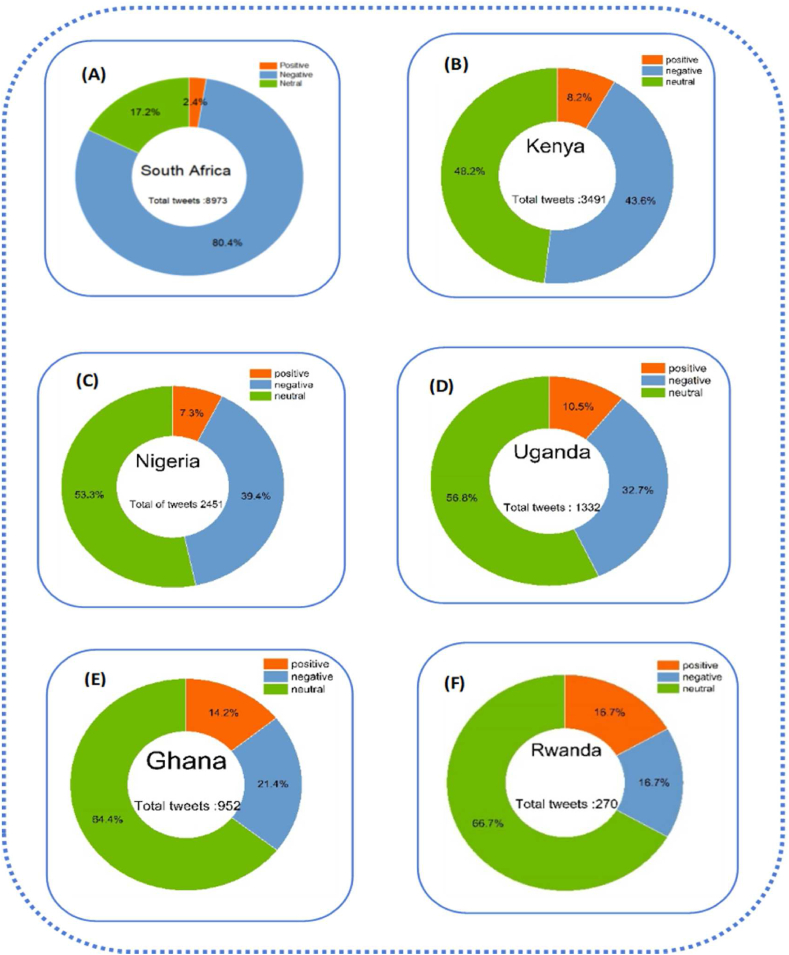
Dispatching global sentiment analysis for six countries in two years. Source: Authors personal data

The result of Fig. 3 shows that among the tweets in our data sets, the proportion of neutral perceptions is higher at, 17.2 % and 48.2 % respectively. The percentage of positive perceptions is lower than the percentage of negative perceptions and the percentage of neutral perceptions, which makes a change in government efforts seen with positive tweets in South Africa 2.4 % and Kenya at 8.2 %. According to these results, we understand that 80 % of tweets in South Africa and 43.6 % of tweets in Kenya are negative. The Ministries of Health have clarified that citizens have to wear a mask in enclosed public places, which remains mandatory for adults and adolescents. South Africa was one of the African countries with the most COVID-19 cases, but it kept its measures strict.

Nigeria presents 7.3 % of positive perceptions, 39.4 % of negative perceptions, and 53.3 % of neutral perceptions, and Ugandan has 10.5 % of positive perceptions, 32.7 % of negative perceptions, and 56.8 % of neutral perceptions. In addition, in Nigeria the fact that officers arrested people for not wearing masks explained the negative perceptions. Since then, Government arrested several people for non-compliance to fight the pandemic. During regulatory measures in places of worship during the rescue operation, both government authorities insisted on the respect of mask-wearing mask wearing In Ghana, 14.2 % of perceptions were positive and 18 % were negative; we found that 66 % of perceptions were neutral; in Rwanda 19 % of positive perceptions, 21.4 % of negative perceptions, and 64.4 % of neutral perceptions. In Ghana and Rwanda, more than half of the reactions were neutral, and in Rwanda, the positive perceptions were higher than the negative perceptions explaining the little trust of citizen for the government. This reason pushes governments to ignore the obligation to wear masks. Also, the percentage shows us how the measures were relaxed compared to the other countries.

In summary, Fig. 3 with A,B, C, D, E and F suggested the most spread sentiments across the neutral and negative perceptions, the context and source of these sentiment figures provide more insight into the factors influencing the sentiment distributions in each country. The percentages highlight variations in the strictness of measures. Rwanda showed higher positive perceptions and relaxed measures compared to other countries.

#### 3.1.2 Lockdown policy

Regarding lockdowns, there are two kinds: while Nigeria and Kenya established full lockdowns, South Africa, Uganda, Ghana, and Rwanda implemented partial lockdowns.

Table 4 provides perceptiveness into the sentiment patterns of Twitter users in these countries over the specified times. The sentiment trends vary between countries, reflecting implicit shifts in public opinion or changes in online discussion.

**Table 4:**
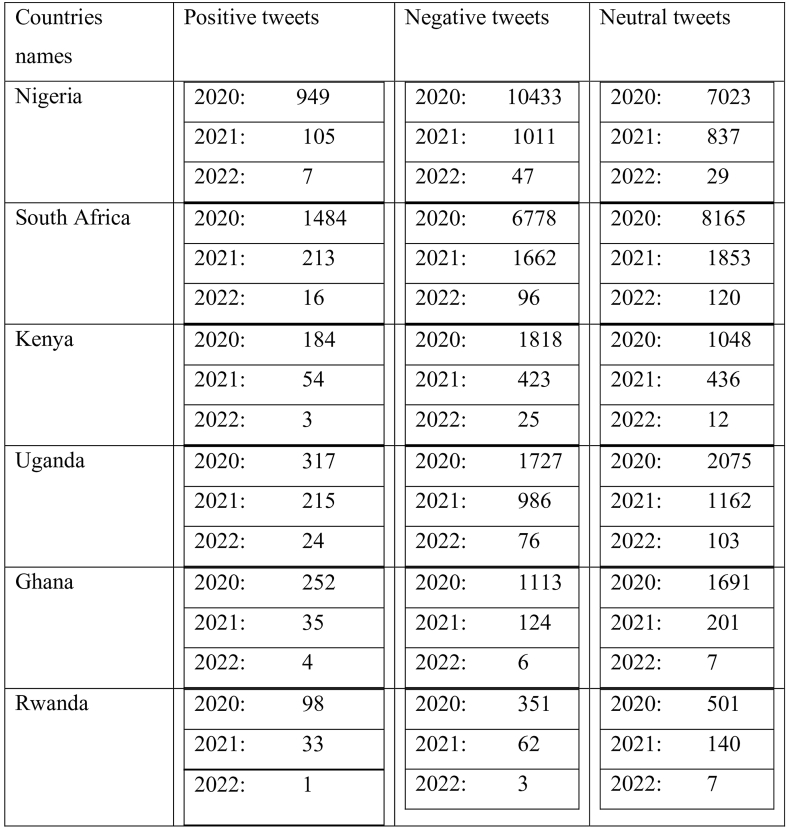
Lockdown sentiment analysis of tweets by country.

Table 5 showed that model which does not appear to be statistically significant, as indicated by the p-value in the ANOVA section. This suggests that the independent variables (lockdown positive tweets, lockdown negative tweets, and lockdown-neutral tweets) may not inclusively have a significant impact on the dependent variable. The coefficients for individual variables are not statistically significant either, indicating that changes in these variables are not reliably associated with changes in the dependent variable in this model.

**Table 5:** Regression Analysis, impact of Lockdown Tweets on Dependent Variable.

Regression Statistics								
Multiple R	0.6064933							
R Square	0.3678341							
Adjusted R Square	0.23237							
Standard Error	0.736109							
Observations	18							
ANOVA								
	*Df*	*SS*	*MS*	*F*	*Significance F*			
Regression	3	4.41400977	1.47133659	2.715362351	0.084508216			
Residual	14	7.58599023	0.541856445					
Total	17	12						
	*Coefficients*	*Standard Error*	*t Stat*	*P-value*	*Lower 95 %*	*Upper 95 %*	*Lower 95.0 %*	*Upper 95.0 %*
Intercept	2021.3206	0.209050629	9669.048284	3.53713E-49	2020.872255	2021.768993	2020.872	2021.769
Lockdown positive tweets.	0.0018777	0.004798616	0.391305338	0.701462391	−0.008414283	0.012169731	−0.00841	0.01217
Lockdown negative tweets.	0.0001434	0.000370273	0.387221741	0.704414753	−0.000650779	0.000937535	−0.00065	0.000938
Lockdown neutral tweets.	−0.0006708	0.001132857	−0.59214282	0.563196583	−0.003100549	0.001758923	−0.0031	0.001759

**Fig. 4:**
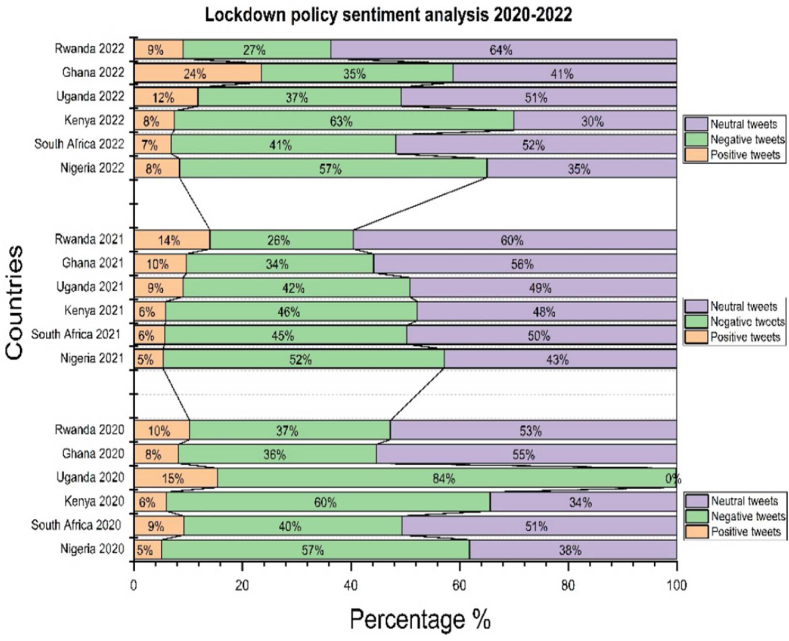
Progressive result for lockdown policy in six African countries from 2020 to 2022.

Fig. 4 explains that lockdowns in Nigeria and South Africa are the countries that presented more tweets than others did; also saw a high pick in these countries in 2020. South Africa has more negative perceptions than neutral ones but Nigeria has more neutral perceptions than negative ones. In 2021, South Africa will have more reactions. Lockdowns in Kenya and Uganda showed more reactions than in Ghana and Rwanda, with the highest pick in 2020. We observed fewer tweets in 2021 and 2022. Kenya showed fewer tweets in 2021, but Uganda showed more. Ghana and Rwanda. We have observed more reactions and fewer neutral opinions than negative ones in 2020. Ghana presented more reactions than Rwanda because, in Rwanda, the government did not put an accent on lockdown policies.

**Fig. 5:**
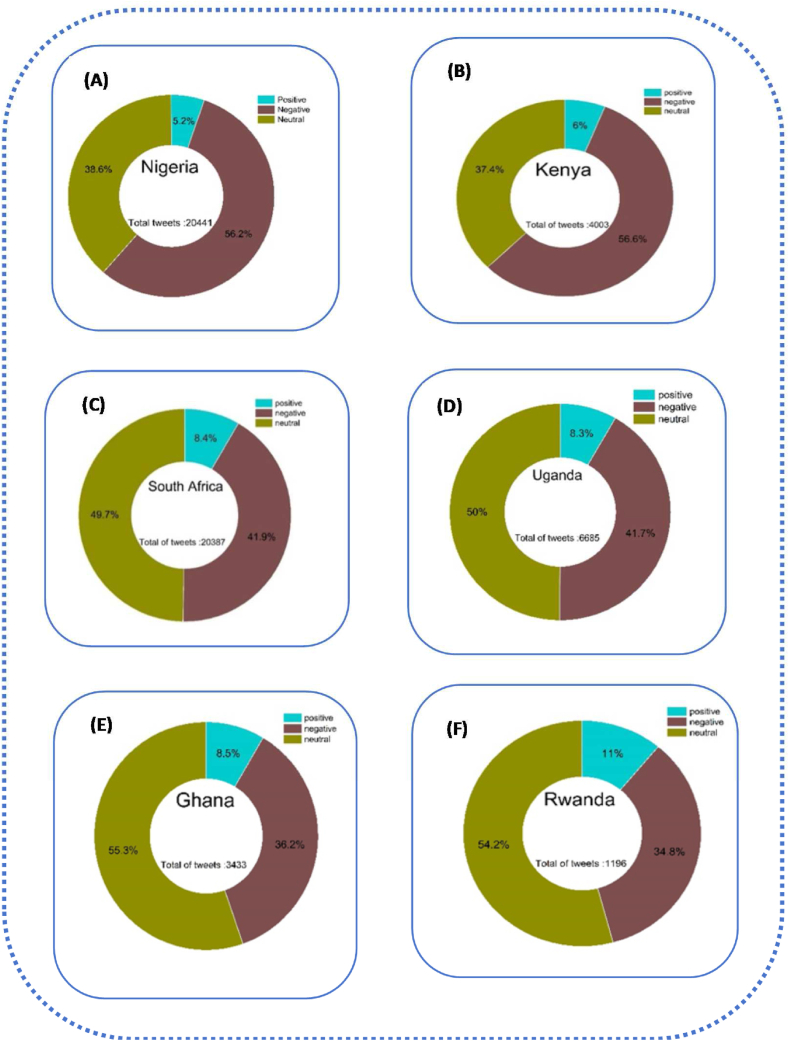
Global result for lockdown policy in six African countries from 2020 to 2022.

Fig. 5 explains that Nigeria and Kenya are on top according to many numbers of negative perceptions compared to other categories. We found in Nigeria 5.2 % of positive perceptions, 56.2 % of negative perceptions, 3.8.6 % of neutral perceptions. Also, in Kenya, 6 % of positive perceptions, 56.6 % of negative perceptions, and 37.4 % of neutral perceptions. Our results clearly showed that Nigeria and Kenya were full and too strict, with a high percentage of negative perceptions. Nigeria a country with a population of 200 million, the president announced the first phase of the lockdown on April 27, 2020, effective from May 4 to May 17, 2020, for two weeks in Lagos. The days were so critical that the governments said if there is not any decision taken, the number of infected people would quickly increase from a few hundred to tens of thousands, and in a few weeks, the lockdown extended to 21 weeks. People who tested positive without being systematically isolated sent to quarantine center. Negative perceptions in Kenya and Nigeria can also be explained by the fact that police officers were deployed everywhere to enforce the measures. When they found citizens on the streets, they were rolling on the floor and doing push-ups with their fists before sent home. Surveillance camera footage showed police chasing people still on the road after curfew hours. In addition, many residents lost their jobs overnight because of a national curfew from 8 p.m. To 6 a.m. Another phase of the lockdown lasted four weeks and began on October 19, 2020.

In contrast, South Africa and Uganda, perceptions were neutral with 49.7 % and 50 % of neutral perceptions, 41.9 % of negative perceptions, and 41.7,8.4 % and 8.3 of positive perceptions. The authorities closed educational institutions; limited gatherings of people outside the workplace to a maximum of 50 people, and restricted sports tournaments, in particular all outdoor activities such as football all amusement parks, restaurants, bars, and nightclubs. Several residents were on partial lockdown. They stayed at home but could still go outside. Respectively, 8.5 % and 11 % of positive perceptions, 36.2 % and 34.8 % of negative perceptions. We found 55.3 % and 54.2 of neutral reaction. The governments of Ghana and Rwanda decided to establish strict lockdowns or anti-pandemic restrictions. They advocated respect for barricade gestures, with a curfew in place from 7p.m Pm to 6a.m. The goal was to make up for the lost time when everyone was imprisoned.

In summary, Fig. 5 showed the severity of lockdown with A and B, strict lockdown with C and D but relaxed lockdown with E and F. The six African countries, expressed the negative perceptions.

#### 3.1.3 Vaccine policy

Regarding vaccines, we found two groups of people concerning the implementation of vaccine policy: on the one hand, citizens who hesitate to take vaccines, and on the other hand, anti-vaccine people. The hesitant are not against vaccination in general but ask themselves or others several questions related to vaccines. Vaccine hesitancy is a global, complex, and diverse process in African countries. Its decryption highlights spiritual, cognitive, or political reasons and the role of the social network.

There are varying patterns in each country's sentiment trends, indicating possible means in public opinion or changes in online conversation in Table 6.

**Table 6:**
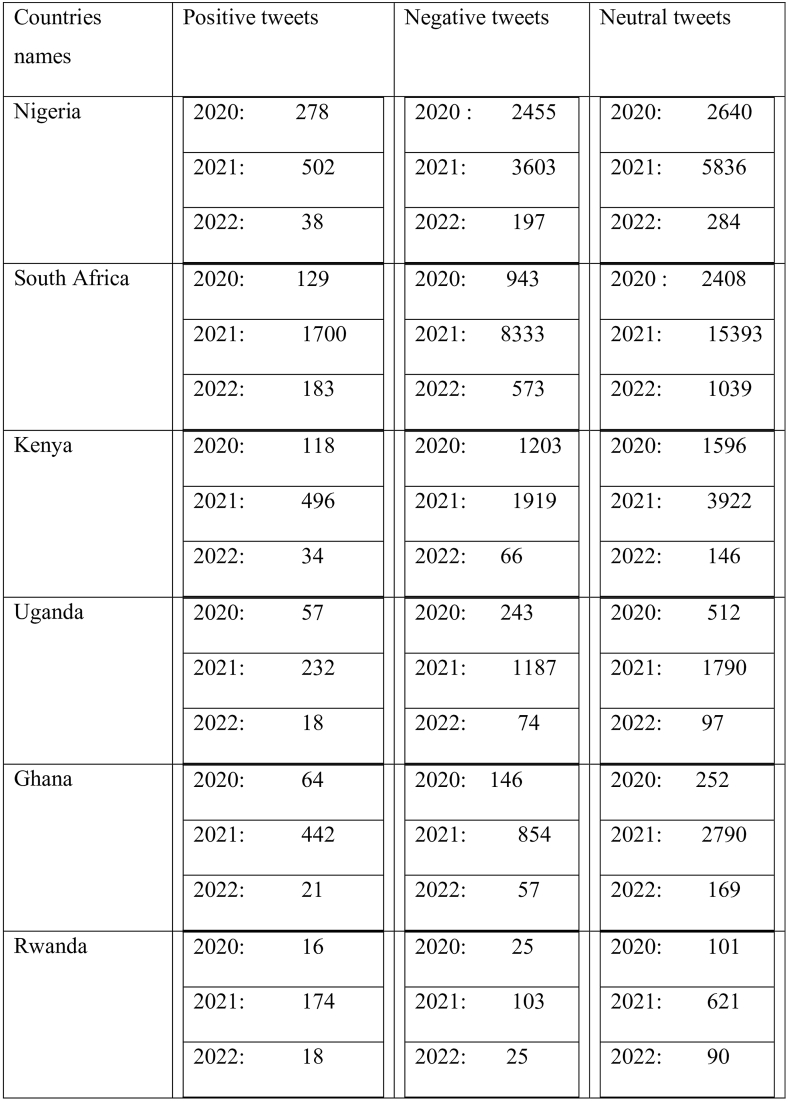
Vaccine sentiment analysis of tweets by country.

Table 7 presents the coefficients for vaccine positive, negative, and neutral tweets representing the change in the independent variable. None of the coefficients for vaccine-related tweets is statistically significant based on their p-values (all above 0.05).

**Table 7:** Regression analysis, impact of vaccine tweets on dependent variable.

Regression Statistics								
Multiple R	0.29804							
R Square	0.088828							
Adjusted R Square	−0.10642							
Standard Error	0.883745							
Observations	18							
ANOVA								
	*Df*	*SS*	*MS*	*F*	*Significance F*			
Regression	3	1.065935	0.355312	0.454942	0.71797			
Residual	14	10.93407	0.781005					
Total	17	12						
	*Coefficients*	*Standard Error*	*t Stat*	*P-value*	*Lower 95 %*	*Upper 95 %*	*Lower 95.0 %*	*Upper 95.0 %*
Intercept	2021.017	0.251517	8035.314	4.72E-48	2020.477	2021.556	2020.477	2021.556
Vaccine positive tweets.	0.002793	0.003423	0.815785	0.428285	−0.00455	0.010135	−0.00455	0.010135
Vaccine negative tweets.	−0.00022	0.000675	−0.32734	0.748255	−0.00167	0.001227	−0.00167	0.001227
Vaccine-neutral tweets.	−0.0002	0.00062	−0.32774	0.747959	−0.00153	0.001126	−0.00153	0.001126

**Fig. 6:**
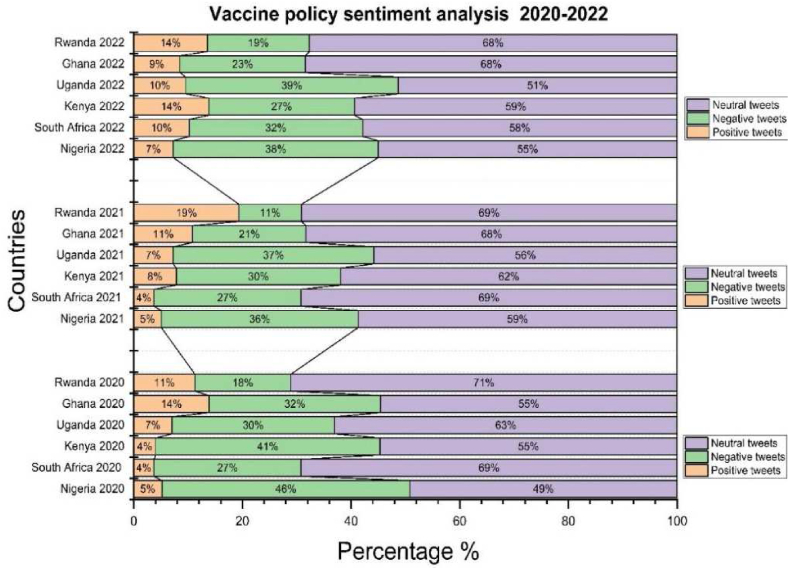
Progressive reactions from 2020 to 2022 in different countries.

Fig. 6 explains vaccine policy reactions in six African countries. We expected a high rate in all countries in 2021. Rwanda was one of the countries with fewer reactions from 2020 to 2022. In 2020 and 2022, we can see the regression of comments in different governments.

**Fig. 7:**
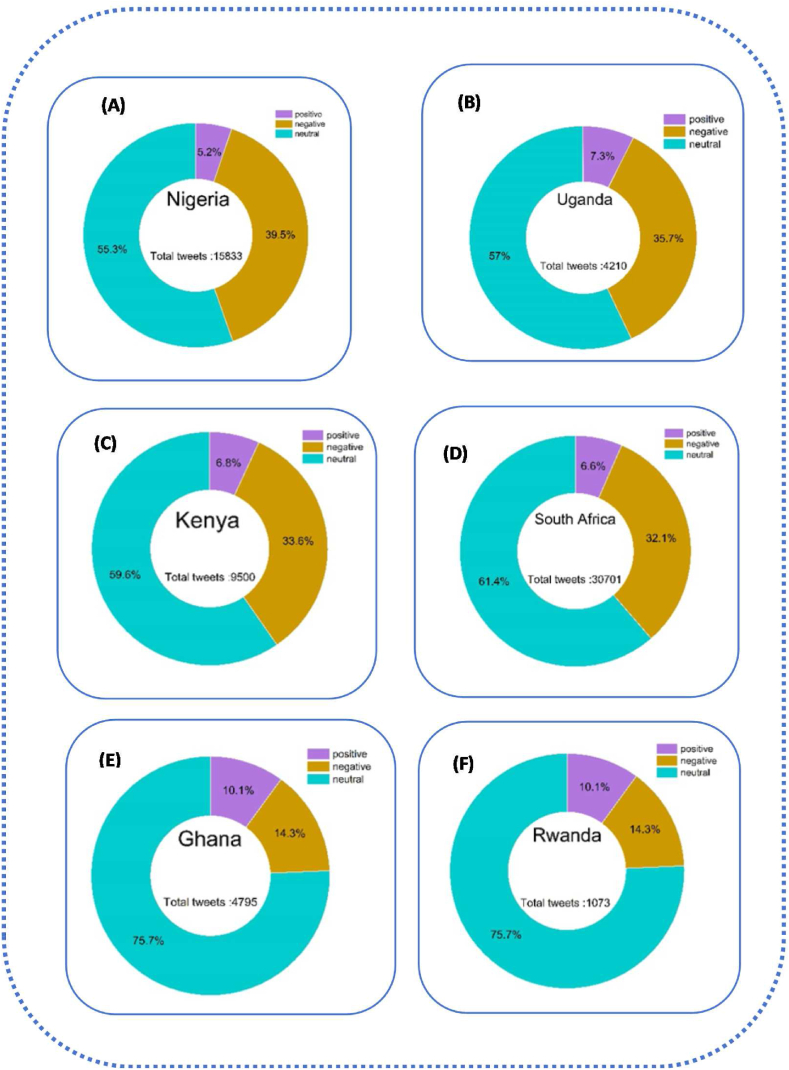
General result of vaccine policies in six African countries From 2020 to 2022.

Fig. 7 clearly shows that Nigeria and Uganda are qualified as two African countries with too strict vaccine measures. As a result, in Nigeria,5.2 % of perceptions were positive, 39.5 % of negative perceptions 55.3 % of neutral perceptions, Uganda with 7.3 %, of positive perceptions, 35.7 % of negative perceptions 47 % of neutral perceptions. The negative perceptions explained the following reasons*:* The hesitation surrounding vaccine acceptance is rooted in concerns about the rapid production of vaccines. Governments have opted to reward individuals with only their salary if they provide proof of vaccination, promotion, or receipt of scholarships. This incentive structure has led to Nigeria having the lowest number of positive tweets, likely influenced by the limited availability of AstraZeneca vaccine doses from the UK, Canada, France, and the United States.

Notably, the World Health Organization (WHO) reported that approximately 50,000 doses of the AstraZeneca vaccine have expired, with Nigeria discarding over one million expired doses. This significant disposal delivery period of vaccines were short, often received less than a month before their expiration.

Moving to Kenya and South Africa, which fall into the second category of vaccine policies, they exhibit 6.8 % and 6.6 % positive perceptions, 33.6 % and 32.1 % negative perceptions, and 59.6 % and 61.4 % neutral perceptions, respectively. In contrast Kenya and South Africa, Ghana and Rwanda implemented relaxed vaccine measures. Leaders in both countries took a proactive approach, actively participating in vaccination campaigns to demonstrate the safety of vaccines. This strategy aimed to dispel fears among those who perceive vaccines as dangerous due to widespread rumours. Despite these efforts, citizens in Ghana and Rwanda did not strongly believe in the necessity of vaccination.

According to governments, the passage of churches and mosques accelerated the vaccination campaign, (Churches and mosques are strategic places where many people flock on days of worship, such as Friday for Muslims and Sunday for Christians). Trust in government was very low in Ghana and Rwanda. We only have between 4 % and 5 % of positive perceptions. Citizens did not agree with the government because it was not generally trustworthy. This is due to less positive perceptions of the management of the COVID-19 vaccine by the government with less than a quarter of the population of respondents agreeing that the management of the pandemic had decreased their trust in the government. Our result shows that more than half of the population reactions were neutral because we looked at the optimal conditions for receiving the vaccine. The fact that the citizens had the choice to react or not can explain the numerous negative perceptions.

In summary, Fig. 7 with A,B,C,D,E and Fsuggest that vaccine measures may lead to hesitancy and negative perceptions, while a middle-ground approach can result in a combination of positive and neutral sentiments. Relaxed measures in Ghana and Rwanda face challenges with low trust and significant neutrality, emphazing the importance of effective communication and public engagement in vaccination compaigns.

### 3.2 Discussion

Our study highlights the impact of policy stringency on public trust in government actions during the COVID-19 pandemic. When governments' implemented policies perceived as either too strict or too relaxed, this suggests that striking a balance between policy measures and effectively communicating the rationale behind these measures is crucial for maintaining public trust. Public perceptions of government policies related to COVID-19, a comparative study in six African countries showed more negative attitudes than positive ones. Personality plays a role in an individual's perceptions of the pandemic situation. On the contrary, most individuals perceived not only negative but also positive aspects of the COVID-19 pandemic situation in 2022 [9].

First, for face mask-wearing policies, the study found that each country's initial face mask-wearing policies had varying levels of negative perceptions. Understanding why certain policies are meeting with negativity could help policymakers improve their messaging and implementation. Public education on the importance of mask wearing and its effectiveness could have influenced these perceptions [10].

Second, concerning lockdowns, the negative feelings in six African countries attributed various factors. These could include concerns about economic hardship, inadequate social support systems, and limited access to essential services during lockdowns. Policymakers need to consider these factors when designing and implementing quarantine measures. Perceived susceptibility and compliance with high levels of perceived susceptibility to COVID-19 and lower compliance, with government measures linked to negative psychological experiences. This highlights the importance of public health campaigns that inform citizens about the risks and provide support mechanisms for those most vulnerable to the virus [11]. Additionally, the economic impact is whether a healthy population can progress in a struggling economy. It underscores the dilemma faced by governments when trying to balance public health measures with economic stability. Addressing the economic concerns of the population through targeted support programs is crucial for gaining public support for pandemic policies. This study highlights that lockdown measures may disproportionately affect those engaged in informal activities. Recognizing the vulnerability of this population and developing policies that address their unique challenges can help alleviate negative perceptions and garner public support. The resumption of increased activity risked increasing the unemployment rate. It is what the governments think about economic growth or respecting the rules of the economy that can also have an impact on the population, a healthy population with a bad economy cannot progress [12]. The government mentioned that the lockdown measures mainly affect the millions of people who live in informal activities and noticed that many of their citizens have an informal situation. COVID-19 is no more threatening than starvation. Even without a lockdown, people live in very difficult conditions that is why the public rejected the lockdown policy because they saw how several households had insufficient access to food [13]. The situation has degraded since the implementation of the lockdown. To fight the coronavirus, if the government imposed too many restrictions, it risked killing the population because of an insufficient supply. The citizens must understand the purpose of their government while they implement strong measures. The group of hesitant people will probably have a role to play and will remain at the center of attention until the end of the race [14].

Third, vaccine policy and religious trust are often rooted in mistrust. In African countries, where religion plays a significant role, the study suggests that religious leaders could be important allies in vaccine promotion. Places of worship have always been anchors for mass vaccination campaigns. It is also because religious leaders and institutions have great power. Religion occupies a preponderant place; it was easier for people to decide to trust their priests than their political leaders. It is part of the African cultural system. According to some pastors and heads of the church, the campaign was their challenge. The vaccine can't protect 100 % against infection, but COVID-19 [15] suggests that building trust within communities through religious institutions and leaders could be an effective strategy for increasing vaccine uptake. Vaccine hesitancy was a significant issue, with concerns about side effects and mistrust of vaccines being major factors. Public health campaigns should focus on dispelling misinformation, providing transparent information about vaccine safety and efficacy, and addressing the concerns of hesitant individuals. Significant factors in vaccine refusal included negative attitudes and poor perceptions towards the anticipated COVID-19 vaccination. Also, Similarly, in a study conducted by Ref. [16] focusing on vaccine hesitancy amongresidents during the COVID-19 pandemic, factors such as conspiracy suspicions, everyday vaccine attitudes, trust, and perceptions of coronavirus were examined as predictors of vaccine hesitancy. The research, which involved an online survey conducted between November 21 and December 21, 2020, concluded that negative perceptions of the vaccine were prevalent. The findings underscore the importance of trust as a crucial factor in increasing the intention of citizen to take vaccine.

This might indicate that the above-mentioned policy strategies did not cover enough target groups. Another potential possibility was that citizens did not have an understanding of the COVID-19 virus from the beginning of 2020–2022. Therefore, continued publicity in the later period did not increase the number of people with positive attitudes. The result clearly showed the intensity of the various sentiments toward the measures implemented by the six African governments. Overall, all six African countries have taken the fight against COVID-19 negatively, and the majority are not in agreement with the government's decisions. On the contrary [17], found positive of citizens who expressed positive opinions according to the policies.

## 4 Conclusion

Public perceptions of government policies toward COVID-19: a comparative study in six African countries from March 07, 2020, to February 28, 2022, by using the Twitter API with different keywords used for scraping data #face# mask# lockdown# vaccine. We used Python sentiment analysis, and we collected 134,494 tweets from citizens' comments on Twitter accounts. The results showed high pick for facemasks, lockdown and vaccine policies in six African countries in 2020, a little regression in 2021, a total regression of tweets in 2022, and a high pick for vaccine policy in 2021. In addition, globally, from March 2020 to February 2022, some countries remained in the same category with all policies like Nigeria and Kenya, South Africa and Uganda with too strcit or strict policies while all the policies in Ghana and Rwanda remained. Overall, we can see that in all six African countries, the majority were not in agreement with the government's decisions, more than half of the reactions were neutral, and a large number of perceptions were negative with all policies. The six countries' sentiment analysis appears to show the governance and economic differences. The COVID-19 pandemic is a matter of life or death for the measures taken as part of the fight against the COVID-19 pandemic. We also found that the continent has one big problem: poverty. First, we suggest that before implementing facemask-wearing and vaccine policies, especially lockdowns, the different governments should check the economy to make sure that the basic facilities are in place for the public to survive during the period. When a large part of the population is in a weak position due to inequality, it means that many will not respect policies and trust the government. We recognize that more than ½ of the populace is dwelling under the poverty line. Second, managing the worldwide border unexpectedly is most necessary to limit the spread.

## Limitations

Obtaining comprehensive data involves various challenges. Here are some difficulties associated with collecting data: accessing data from social media platforms, such as Twitter, is subject to API limitations and restrictions. The platforms may limit the volume of retrieved data, and historical data access might be constrained. Collecting, cleaning, and analysing large volumes of social media data is resource-intensive. It requires computational resources, expertise in data analytics, and careful consideration of methodological approaches. In addition, translating information from tweets to represent the totality of responses of the entire population of each of the six countries regarding vaccine, lockdown, and face mask. If policies involve several limitations, it means that the number of tweets collected may not be proportionate to the population size of each country. For some countries, especially those with fewer tweets, including the entire population's sentiments becomes more challenging. Social media usage varies across countries, regions, and demographic groups. Obtaining a balanced dataset that represents the diversity of social media engagement across the studied countries is challenging. As observed through sentiment analysis of tweets, there is the potential issue of limited representation. While social media platforms offer a valuable window into public discourse, it is essential to acknowledge that the number of citizens who actively engage in tweeting represents only a fraction of the overall population. Although the sentiments expressed on social media may not comprehensively reflect the views of the entire population of the respective countries under examination, the exclusion of non-internet users means that the analysis of tweets inherently excludes individuals who do not have access to the internet or who choose not to use social media platforms. This exclusionary aspect of the data may overlook the opinions and concerns of significant segments of the population, particularly in regions with limited internet penetration. Moreover, demographic disparities mean that social media usage varies across age groups and demographics. Younger individuals tend to be more active on social media, potentially resulting in a skewed representation of generational perspectives. It may not fully capture the sentiments of older or less tech-savvy citizens.

## Implications

To address the limitation of limited representation on social media, future studies should consider incorporating data from complementary sources, such as surveys, interviews, or focus groups. These methods can provide a more comprehensive understanding of public perceptions and include voices that are not present on social media. Qualitative analysis to delve deeper into the motivations, concerns, and experiences of individuals, providing a richer context for understanding public sentiment. Combining quantitative sentiment analysis with qualitative research can offer a more holistic perspective. Policymakers should recognize that while social media provides valuable insights, decisions based solely on social media sentiment might not adequately represent the needs and concerns of all citizens. Engaging in a broader consultation process that includes diverse voices is essential for robust policy development. In conclusion, while sentiment analysis of tweets offers a valuable snapshot of public sentiment, it is essential to acknowledge its limitations, particularly the issue of limited representation. Researchers and policymakers should exercise caution and consider complementary data sources and methods to ensure a more comprehensive and accurate understanding of public perceptions in the context of government policies related to COVID-19; also, investigate how government policies during the pandemic may have affected the environment, such as changes in pollution levels, waste management, and conservation efforts. Future researchers can extend the analysis by collecting netizens’ perceptions of government policies during the COVID-19 pandemic.

## Funding

The Chinese Ministry of Education of Humanities and Social Science Project, Grant number 23YJAZH147, supports this work.

## Data availability statement

The data that supports the findings of this study is available. In this paper, we retrieved data from Twitter API and coded in Python software.

https://colab.research.google.com/drive/1DBfJHpx81g160E9ziJndtWwPrLkEvKgc?usp=shari(Datascraping);

https://colab.research.google.com/drive/18gkDeb-UvtoZmZLGqjmnWoyFgYWGlypT?usp=sharing (Data cleaning);

https://colab.research.google.com/drive/1y22pV5BMuNqIo548GjTbBYnrBCLJ3JE?usp=sharing (Sentiment analysis).

## CRediT authorship contribution statement

**Yi-jun WANG:** Validation, Supervision, Methodology, Funding acquisition, Conceptualization. **Marly Loria DIABAKANGA BATATANA:** Writing – review & editing, Writing – original draft, Resources, Investigation, Formal analysis. **Maximino Horacio BIKOUMOU GAMBAT:** Visualization, Software, Data curation.

## Declaration of competing interest

The authors declare the following financial interests/personal relationships which may be considered as potential competing interests:Yi-jun WANG, Marly Loria DIABAKANGA BATATANA and MaximinoHoracio BIKOUMOU GAMBAT reports financial support was provided by The Chinese Ministry of Education of Humanities and Social Science Project, Grant number 23YJAZH147. If there are other authors, they declare that they have no known competing financial interests or personal relationships that could have appeared to influence the work reported in this paper.

## References

[1] Pokhrel S., Chhetri R. (2021). A literature review on impact of COVID-19 pandemic on teaching and learning. Higher Edu. Future.

[2] Zhou F., Yu T., Du R., Fan G., Liu Y., Liu Z., Xiang J., Wang Y., Song B., Gu X., Guan L., Wei Y., Li H., Wu X., Xu J., Tu S., Zhang Y., Chen H., Cao B. (2020). Clinical course and risk factors for mortality of adult inpatients with COVID-19 in Wuhan, China: a retrospective cohort study. Lancet.

[3] Albzeirat M., Zulkepli N., Qaralleh H. (2022). A vision to face covid-19 pandemic and future risks through artificial intelligence. Jbarbiomed.

[4] Dennison Himmelfarb C.R., Baptiste D. (2020). Coronavirus disease (COVID-19): implications for cardiovascular and socially at-risk populations. J. Cardiovasc. Nurs..

[5] Choi B., Choudhary M.C., Regan J., Sparks J.A., Padera R.F., Qiu X., Solomon I.H., Kuo H.-H., Boucau J., Bowman K., Adhikari U.D., Winkler M.L., Mueller A.A., Hsu T.Y.-T., Desjardins M., Baden L.R., Chan B.T., Walker B.D., Lichterfeld M., Brigl M., Kwon D.S., Kanjilal S., Richardson E.T., Jonsson A.H., Alter G., Barczak A.K., Hanage W.P., Yu X.G., Gaiha G.D., Seaman M.S., Cernadas M., Li J.Z. (2020). Persistence and evolution of SARS-CoV-2 in an immunocompromised host. N. Engl. J. Med..

[6] Chen E., Ferrara E. (2023). Tweets in time of conflict: a public dataset tracking the twitter discourse on the war between Ukraine and Russia. ICWSM.

[7] Khan S., Umer R., Umer S., Naqvi S. (2021). Antecedents of trust in using social media for E-government services: an empirical study in Pakistan. Technol. Soc..

[8] Bellar O., Baina A., Bellafkih M., Hassanien A.E., Haqiq A., Azar A.T., Santosh K., Jabbar M.A., Słowik A., Subashini P. (2023). The 3rd International Conference on Artificial Intelligence and Computer Vision (AICV2023), March 5–7, 2023.

[9] Fadillah R. (2019). STUDENTS’ perception on the use of mind mapping application software in learning writing. Celtica.

[10] Wang S., Han C., Sang Z., Zhang X., Chen S., Wang H., Wang G., Xu Y., Lei X., Chen J. (2023). Hidden faces, altered perceptions: the impact of face masks on interpersonal perception. Front. Psychol..

[11] Commodari E., La Rosa V.L., Carnemolla G., Parisi J. (2021). The psychological impact of the lockdown on Italian university students during the first wave of COVID-19 pandemic: psychological experiences, health risk perceptions, distance learning, and future perspectives. Mediterranean J. Clinical Psychol..

[12] Aditya B., Amri I. (2023). Rethinking informal economy resilience during crisis: experience from COVID-19 pandemic. Indian J. Lab. Econ..

[13] Grunert K.G., De Bauw M., Dean M., Lähteenmäki L., Maison D., Pennanen K., Sandell M.A., Stasiuk K., Stickel L., Tarrega A., Vainio A., Vranken L. (2021). No lockdown in the kitchen: how the COVID-19 pandemic has affected food-related behaviours. Food Res. Int..

[14] Abdeljaoued-Tej I. (2020). A pandemic at the Tunisian scale. Mathematical modelling of reported and unreported COVID-19 infected cases. Epidemiology.

[15] Adane M., Ademas A., Kloos H. (2022). Knowledge, attitudes, and perceptions of COVID-19 vaccine and refusal to receive COVID-19 vaccine among healthcare workers in northeastern Ethiopia. BMC Publ. Health.

[16] Dupont C., Oberthür S., Von Homeyer I. (2020). The Covid-19 crisis: a critical juncture for EU climate policy development?. J. Eur. Integrat..

[17] Bakul F., Heanoy E.Z. (2022). Impact of COVID-19 anxiety on loneliness and sleep quality of students and professionals in Bangladesh. Acta Psychol..

